# Genomic characterization of Nontuberculous Mycobacteria

**DOI:** 10.1038/srep45258

**Published:** 2017-03-27

**Authors:** Tarcisio Fedrizzi, Conor J. Meehan, Antonella Grottola, Elisabetta Giacobazzi, Giulia Fregni Serpini, Sara Tagliazucchi, Anna Fabio, Clotilde Bettua, Roberto Bertorelli, Veronica De Sanctis, Fabio Rumpianesi, Monica Pecorari, Olivier Jousson, Enrico Tortoli, Nicola Segata

**Affiliations:** 1 Centre for Integrative Biology, University of Trento, Trento, Italy; 2 Mycobacteriology unit, Department of Biomedical Science, Institute of Tropical Medicine, Antwerp, Belgium; 3 Microbiology and Virology Unit, University Hospital Polyclinic, Modena, Italy; 4 NGS Facility, Laboratory of Biomolecular Sequence and Structure Analysis for Health, Centre for Integrative Biology, University of Trento, Italy; 5 Emerging Bacterial Pathogens Unit, IRCCS San Raffaele Scientific Institute, Milano, Italy

## Abstract

*Mycobacterium tuberculosis* and *Mycobacterium leprae* have remained, for many years, the primary species of the genus *Mycobacterium* of clinical and microbiological interest. The other members of the genus, referred to as nontuberculous mycobacteria (NTM), have long been underinvestigated. In the last decades, however, the number of reports linking various NTM species with human diseases has steadily increased and treatment difficulties have emerged. Despite the availability of whole genome sequencing technologies, limited effort has been devoted to the genetic characterization of NTM species. As a consequence, the taxonomic and phylogenetic structure of the genus remains unsettled and genomic information is lacking to support the identification of these organisms in a clinical setting. In this work, we widen the knowledge of NTMs by reconstructing and analyzing the genomes of 41 previously uncharacterized NTM species. We provide the first comprehensive characterization of the genomic diversity of NTMs and open new venues for the clinical identification of opportunistic pathogens from this genus.

The genus *Mycobacterium* is a homogeneous taxonomic entity whose members have many distinguishing phenotypic and genotypic features separating them from other genera. Mycobacterial species populate a diverse set of natural and human-associated environments^1^. Despite the fact that more than 170 species are officially recognized within the genus *Mycobacterium*, the research has mainly focused so far on obligate pathogen species, and in particular on *M. tuberculosis*^2^. The members of the *M. tuberculosis* complex are responsible for tuberculosis which is a leading cause of death worldwide^3^ and it is thus unsurprising that a very large number of *M. tuberculosis* strains have been sequenced, producing by far many more genomes than all the other mycobacterial species together. The analysis of the genomic features of such strains were functional to elucidate outbreak dynamics at very high genetic resolution^4^,^5^,^6^ and to identify genomic determinants of drug resistance and virulence^7^,^8^.

Nontuberculous mycobacteria (NTM) include all *Mycobacterium* species that do not cause tuberculosis or leprosy, thus excluding the species of the *M. tuberculosis* complex as well as *M. leprae* and “*M. lepromatosis”*. Among NTM species, an increasing number of strains have been deemed responsible, in the last decades, of severe and treatment-resistant diseases^9^,^10^,^11^,^12^,^13^. NTMs are present in the environment, in particular in water and soil^14^, and can occasionally infect humans or animals causing a range of pathological conditions including pulmonary, skin, bone, joint, and disseminated diseases in the presence of various predisposing conditions (primarily chronic diseases and immunosuppression)^15^,^16^,^17^,^18^. Although several NTM species are now recognized as a major infective threat^19^, their in-depth genomic investigation has not been carried out systematically. For example, it is unknown whether characterized virulence factors occurring in *M. tuberculosis* and in the most studied NTM species, including proline-glutamate/proline-proline-glutamate motif proteins (PE/PPE), the ESX export systems, the mammalian cell entry (Mce) protein family, the Sec-dependent general secretion system and the Twin-arginine translocase (Tat) export system^20^,^21^,^22^,^23^,^24^, are widespread or not in the many NTM species without available genomic information. Given the current availability and cost-effectiveness of whole-genome sequencing (WGS) approaches, we aimed to characterize the diversity within the *Mycobacterium* genus and investigate the phylogenetic relationships and functional potential of many poorly known mycobacterial species.

The few comprehensive phylogenetic analyses of the whole genus *Mycobacterium* have been based so far primarily on the comparison of single^25^ or concatenated housekeeping genes^26^. The 16S rRNA gene has been the most used marker and the topology of the phylogenetic trees based on its sequences are substantially in agreement with those emerging from the multilocus sequencing approach. A recent whole genome phylogenetic analysis of 40 *Mycobacterium* strains confirmed previous results^27^. However, it is unknown whether the presently accepted phylogeny, characterized by several groupings of closely related species, remain consistent when considering many NTM species that are still not sequenced. The *M. tuberculosis* complex, the *M. avium* complex, and the *M. terrae* complex are the best-known groups, but others complexes have been defined and named using a representative species (*M. abscessus, M. celatum, M. fortuitum, M. kansasii, M. marinum, M. simiae* and *M. smegmatis*). The current definition of such groups is mainly based on the presence of genetic signatures at the level of 16S rRNA gene and, to a lesser extent, on shared phenotypic and epidemiologic characteristics.

In this work, we thus aimed at filling the gaps in the current genomic and phylogenetic characterization of the *Mycobacterium* genus and at providing the basis for the search of new determinants of mycobacterial virulence and antibiotic resistance. To this end, we sequenced and assembled the genome of 41 new NTM species. By analyzing these genomes in the context of the few dozens of already sequenced mycobacterial species, we were able to substantially increase our knowledge on the genetic and functional diversity within this genus, to provide the foundations of an ultimate phylogeny and to assess the evolutionary impact of horizontal gene transfer.

## Results and Discussion

### Sequencing and assembly of 47 NTM genomes

In order to investigate the genomic features of NTMs we targeted for sequencing 44 type strains of different *Mycobacterium* species along with three previously described clinical strains^28^. Representative species of both rapid and slow growing mycobacteria were included (11 and 36 strains respectively) with preference for those species without available genome in public databases. Particular focus was given to the species known to be members of phylogenetically related clades on the basis of 16S rRNA sequences.

The genomes were sequenced using the Illumina platform (100 nt paired-end reads, see Methods) generating a total of 70 Gb with an average coverage of 61X per genome. Genomes were assembled using SPAdes^29^ and high-quality assemblies were obtained for 44 of the 47 genomes belonging to 41 species (see Methods). The three species for which we were not able to obtain a high quality assembly were *M. neworleansense, M. mantenii*, and *M. heraklionense*. We obtained on average 93 (s.d. 58) contigs per genome corresponding to an average N50 score of 192 kb (s.d. 148 kb) with reduced variability across mycobacterial complexes as reported in Table 1 and Supplementary Table 1.

The species of the *M. terrae* complex^30^ were characterized by short genomes (avg. 4.5 Mb, s.d. 0.2 Mb) and by the highest GC content (avg. 68.3%, s.d. 0.4%). The shortest genome we sequenced is *M. triviale* (3.6 Mb). In contrast, *M. gordonae* had a genome of 7.6 Mb, one of the longest in the genus (99.8 quantile) and in the bacterial kingdom (97.5 quantile, the longest being *Sorangium cellulosum* 14.8 Mb)^31^. Open reading frames (ORFs) were identified with the Prokka pipeline^32^ (see Methods). The samples with the highest and lowest number of ORFs were *M. wolinskyi* and *M. triviale*, respectively. GC content was also remarkably homogeneous at the complex level (Table 1) with only the *M. simiae* complex showing a greater standard deviation. ORFs and genome lengths were indeed consistent across the complexes as confirmed by low standard deviations (Table 1).

### Phylogenetic analysis supports and improves the categorization of monophyletic and paraphyletic mycobacterial complexes

**Figure 1:**
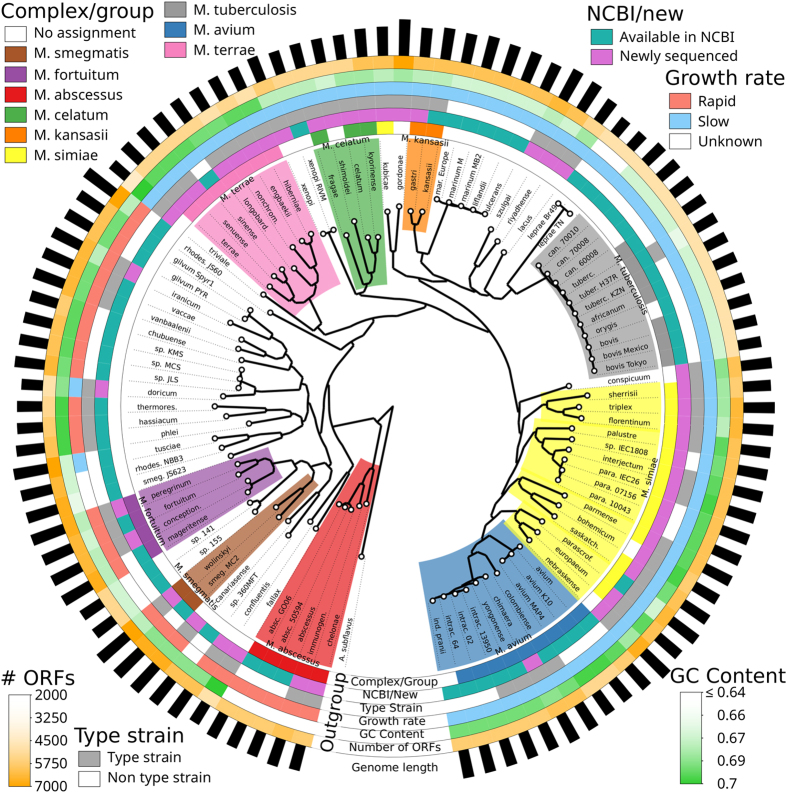
Whole-genome phylogeny of the Mycobacterium genus reconstructed using the newly sequenced genomes and the ones that were already available. The tree is built using the concatenated alignments of the 243 fully conserved genes within the genus with the maximum-likelihood inference approach implemented in RAxML^65^ (see Methods) and displayed using GraPhlAn^74^. Colored shades highlight the Mycobacteria groups/complexes including the newly inferred assignments supported by the phylogeny. External to the phylogeny, we annotate the original group assignments and, for each strain, whether it is a newly sequenced or already sequenced strain, whether it is a type strain, its growth rate, its average GC content and the number of identified ORFs. The lengths of the outer black bars are proportional to the total genome length. The abbreviations used in this figure are reported in Supplementary Table 2.

In order to investigate the evolutionary history of mycobacteria, we reconstructed the phylogeny of the genus using a concatenated core genome approach (Fig. 1 and Supplementary Figure 1, abbreviations listed in Supplementary Table 2). Both already available and newly sequenced strains were included (99 isolates in total, see Methods) and *Amycolicicoccus subflavus* (family Mycobacteriaceae) was used as outgroup as it is the sequenced bacterium closest to the *Mycobacterium* genus. The comparison of the phylogenetic trees built on the core genomes and on the 16 S rRNA sequences revealed a substantial concordance (distance of 44 rSPR^33^ - the minimum number of topology changes needed to reconcile two trees^34^). In both phylogenies, rapid growers were clearly separated from slow growers with the *M. terrae* complex occupying an intermediate position. Rapid growers appeared more ancestral and the phylogeny suggested that there is a common ancestor for the whole genus, most closely related to the present *M. abscessus* complex. Among rapid growers, three monophyletic groups coincide with the *M. abscessus, M. fortuitum* and *M. smegmatis* complexes. Two slowly growing species, *M. doricum* and *M. tusciae*, branch among rapid growers; interestingly, a similar misplacement was highlighted in 16S rRNA-based phylogenetic analysis. However, the *M. tusciae* isolate with an already available genome (strain JS617) has a full-length 16S rRNA similarity with that of *M. tusciae* type strain (CIP106367) that questions its taxonomic label. Complete agreement was displayed by the *M. terrae* complex that is unambiguously defined in the conventional phylogeny, by a 14 nucleotide insertion in the helix 18 of its 16S rRNA gene. Intriguingly, the newly sequenced *M. triviale* branches basally to the *M. terrae* complex supporting the hypothesis that this rapid grower might represent the link in the evolutionary pathway leading towards the slowly growing species.

Among the slow growers, several monophyletic groups included the species known to be related to *M. celatum, M. kansasii, M. marinum, M. tuberculosis* and *M. avium*. The *M. simiae* complex, which traditionally includes all the slowly growing species sharing the short helix 18 signature in the 16S rRNA^35^, revealed instead to be paraphyletic with respect to the *M. avium* complex, with at least four genomically well defined subgroups. This finding is in agreement with diversified phenotypes of the species of the complex which present different colony pigmentation and different cell wall mycolic acid patterns^36^.

The phylogenetic analysis of core genomes also highlights potential conflicts in the taxonomy and suggests new group assignments. For instance, the traditional inclusion of *M. kubicae* in the *M. simiae* complex^25^ is not supported by genomic phylogeny (the ANI score between *M. kubicae* and the closest *M. simiae* strain is 78.8%). Conversely, our study provided evidence for the previously unknown inclusion of *M. shimoidei* in the *M. celatum* group (ANI score of 81.3% between *M. shimoidei* and the closest *M. celatum* strain), and of both *M. bohemicum* and *M. nebraskense* in the *M. simiae* complex (closest ANI scores of 82.5% and 87.5 respectively). The expanded taxonomy was also helpful in detecting inconsistencies of the labelling of already available genomes. Strain JS623^37^, for example, although labelled as *M. smegmatis*, was clearly outside of the phylogenetic branch leading to the latter species and is unquestionably an uncharacterized mycobacterium other than *M. smegmatis*, as was also confirmed by looking at the sequences of most housekeeping genes (data not shown). JS60 and NBB3, both labelled as *M. rhodesiae*, were phylogenetically very distant and both substantially differing from the type strain of the species.

### The newly sequenced genes double the genomic diversity in the genus and confirm the phylogenetic relations

**Figure 2:**
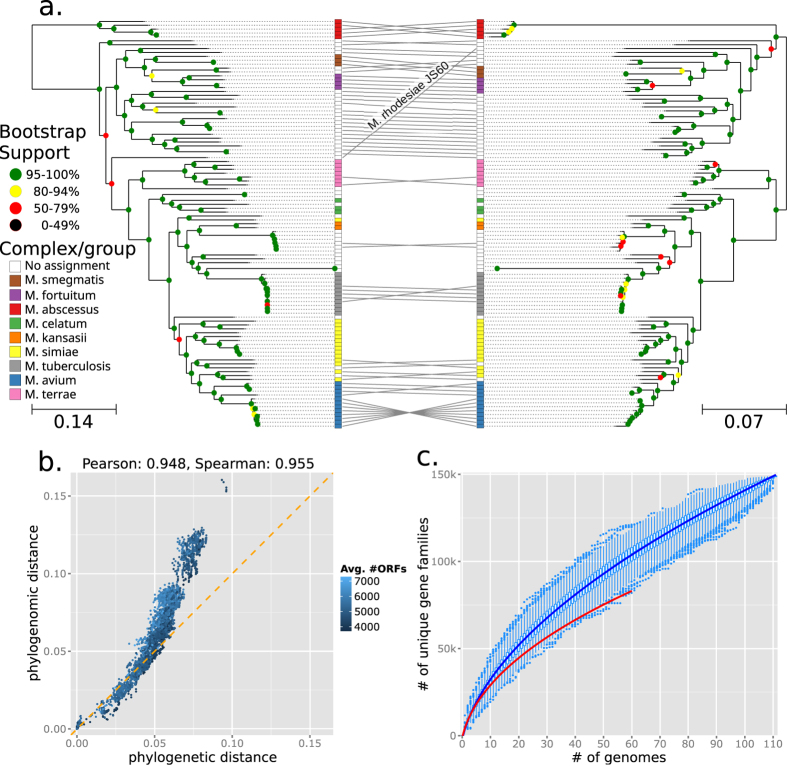
Comparison of genome relations as inferred by the sequence-based phylogeny and the gene presence/absence clustering and the size of the non-redundant gene catalog of the Mycobacterium genus. (**A**) The phylogenetic tree (on the left) and the gene presence/absence tree (on the right) are contrasted to highlight the consistency of the complementary evolutionary signals and identifying taxa with potentially uncoupled genetic versus functional evolution. (**B**) The scatter-plot of the pair-wise distance of the strains in the phylogenetic versus gene presence/absence trees (color denotes the average number of ORFs between the compared strains). Both the arrangement of the points and the overall correlation support the consistency between the trees. (**C**) The increase of the pan-genome size as a function of the number of genomes included in the clustering (see Methods). The blue curve highlights the trend for all the genomes (newly sequenced and retrieved from NCBI), whereas the red curve refers to already available genomes only. This analysis suggests that genomes sequenced in this work roughly double the gene families available for the Mycobacterium genus as also confirmed by other clustering approaches (see Methods).

By sequencing the genomes from 41 new NTM type strains we greatly enhanced the genomic characterization of the *Mycobacterium* genus. The pan-genome built by binning genes with a sequence similarity >80% (see Methods) almost doubles, expanding to 150 thousand unique gene families, the potential functional repertoire of mycobacteria. Although the absolute size of the pangenome is dependent on the gene identity threshold considered, the application of another available gene clustering approach^38^ confirmed the 2X expansion of the number of pan-genes when considering the newly sequenced genomes (see Methods). Importantly, the genus presents an open pan-genome (Fig. 2C) which implies that, with other known species still unsequenced and with many additional species still unknown, a higher set of divergent functions are expected. This also confirms the highly diverse gene content of mycobacteria.

The hierarchical clustering tree constructed on the basis of the gene families’ presence/absence pattern (Supplementary Figure 2) confirms that all the complexes are monophyletic except for the *M. simia*e group. By directly contrasting the results obtained with approaches based on core genome and gene presence/absence we aimed to highlight the mismatches between divergent function and genetic evolution (Fig. 2A). The only case of substantial disagreement was *M. rhodesiae* strain JS60 which is however placed with very low bootstrapping support by both approaches. The high consistency between functional specialization and baseline genetic divergence (0.95 correlation coefficient, Fig. 2B) suggests a low impact of horizontal gene transfer events for mycobacteria and a low frequency of rapid functional adaptation cases.

### Gene annotation analysis highlights functional specialization of mycobacterial complexes

**Figure 3:**
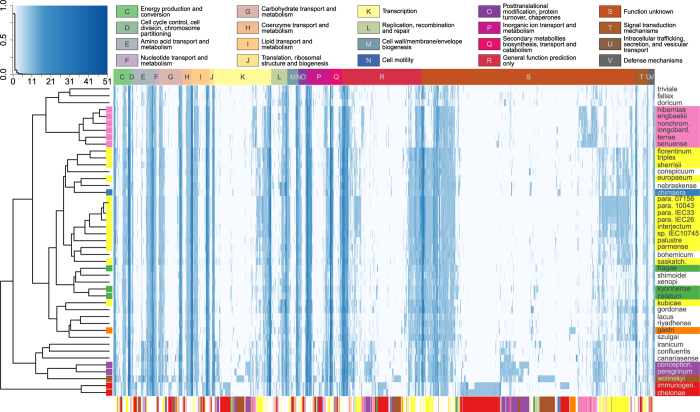
Heatmap representing the distribution of EggNOG functions within the newly sequenced genomes. The heatmap reports the number of genes that are labeled with a particular function within each sample we sequenced. The horizontal top bar represents the functional category. Colors in the bar at the bottom indicate the genes specific for a particular complex (fisher test p < 0.05). The full names for the abbreviated species names are reported in Supplementary Table 2.

We functionally annotated the genomes using the eggNOG database^39^ (Fig. 3). On average, 61% of the genes of a genome were assigned to poorly functionally characterized categories (R - “General function prediction only” and S - “Function unknown”), had no definitive function assigned, or no homologue was found in the eggNOG database. The remaining annotations primarily assigned genes to transcription (category K; ~4.7%/genome), lipid transport and metabolism (category I, ~4.2%/genome) and energy production and conversion (category C, ~3.52%/genome) (Supplementary Table 3). The hierarchical clustering based on the presence/absence of functional categories is again very consistent with the other phylogenetic trees constructed for this group. Monophyletic complexes remain indeed well separated and the fragmentation of the *M. simiae* complex reflect its highly heterogeneous composition.

Several genes were found to be specific of a given complex. On average, 420 genes were present in a specific complex and absent from all others (range: 51–731; Supplementary Table 4). Categorisation of these clade-specific genes confirmed that, similar to the overall genome patterns, 61% had unknown or poorly characterized functions. Discounting these, most other discriminating genes belonged to the transcription COG category (category K, ~11%/genome), indicating that the complexes are largely distinguished from each other in the way they regulate the expression of their functional repertoires. Discriminating functions were further searched for operon-like patterns: any genes that were found to be within 2 genes from another discriminating gene were labeled as a likely operon. Large clusters of genes (more than 5 co-localised) were found in the *M. abscessus* group (3 clusters) and *M. fortuitum* (1 cluster) complex (Supplementary Table 5). However, the high number of hypothetical and broadly characterized proteins in these clusters makes distinguishing their specific functions difficult.

### Low abundances of mobile elements were observed in newly sequenced species

Plasmids and (pro)phages can be crucial genomic features connected with relevant phenotypic and clinical outcomes. We thus implemented a set of procedures for *de novo*, and mapping-based, discovery of plasmid and phage sequences in the assemblies (see Methods). Overall, 26 potential plasmids/phages (3 by sequence mapping, 23 by read depth analysis) were found within the dataset, spread across 9 strains (Supplementary Table 6). These mobile elements were found in three *Mycobacterium* complexes, with similar numbers of mobile elements per complex, and in 4 ungrouped species (Table 2). Nine of these contigs were found to contain either only transposases or transposases plus another gene, suggesting the occurrence of transposable elements within the chromosome. Three contigs within the same genome were found by homology search to known plasmids (strain 768; *M. chimaera*) and all appeared to be the plasmid pMK12478 derived from *M. kansasii* (86% average nucleotide identity), suggesting a potential horizontal transfer between species belonging to unrelated complexes. Several contigs covered different portions of the plasmid, suggesting a single plasmid that had not been recovered as a whole during sequencing.

For 14 contigs containing mobile elements without reliable homology in the NCBI sequence repositories, we sought evidence supporting the prediction of being true plasmids or phages. However, confirmation of plasmid-related sequences using functional annotations^40^,^41^ was not possible due to lack of specific markers for such mobile elements. We further sought for confirmation of the presence of phages within each genome, but no significant full-genome homology was found between these isolates and a known *Mycobacterium* phage. Phage-related annotations were found in six of 26 potential plasmid/phage contigs, all of which were not labelled as likely transposases, although the promiscuity of such annotations within genomes cannot provide complete confirmation. Functional annotation of the 26 putative mobile element contigs resulted in an overwhelming number of hypothetical proteins (54.5% compared to 17.8% in the genomes) confirming the functional specialization of extra-chromosomal and mobile elements. Additional searching for transposes based on eggNOG annotations also revealed low abundances across the genomes (Supplementary Table 7), ranging from 0.11% to 1.81% of genome contents.

Altogether, our analysis suggests that it is likely that several circular, potential mobile elements portions are present in the genomic repertoire of many *Mycobacterium* genomes. Although many of these appear to likely be phages, further sequencing efforts are required to confirm both their identity and function.

### Lateral gene transfers have a low impact on genomic evolution of mycobacteria

Analysis of putative lateral gene transfer (LGT) events revealed low levels of LGT in all genomes as already suggested by the comparison between functional and phylogenetic evolution. The percentage of genomes resulting from LGT ranged from 0.04% to 1.9% (Table 3 and Supplementary Table 8). No LGT-derived genes were found on predicted phage/plasmid contigs, suggesting that these mobile elements may be species-specific and do not result in largely altered gene repertoires. Additionally, between-NTM recombination analysis of the core genes revealed no large (>1 kb) events, which is also indicative of no other large recombinations as these genes are spread through the genomes of each species. These results suggest that LGT has very little impact on mycobacterial genomes, perhaps even lower than has been previously reported^42^. The potential donors of these LGT-derived genes were found to derive from over 130 genera, primarily from the Actinobacteria and a small number of Beta-proteobacteria. Five genera (*Amycolatopsis, Frankia, Nocardia, Rhodococcus* and *Streptomyces*) were highlighted as potential donors in over half the species, indicating close relationships with organisms belonging to the same class (Actinobacteria) as mycobacteria. As members of these genera often share a similar environment (primarily soil-dwelling) it seems likely that most LGT is habitat-driven.

The large majority of genes acquired through LGT were annotated as genes of unknown function (eggNOG categories R and S and ‘none’, Supplementary Figure 3). The second most frequently transferred category were proteins involved in energy production and conversion. Many oxidoreductases were found to be transferred in addition to many other reductases and dehydrogenases. Some genomes also acquired carbohydrate and amino acid metabolism and transport genes (categories G and E respectively), and primarily membrane transporters for both. These findings suggest that LGT resulted in increased capabilities of growth and nutrient transport, likely allowing for habitat adaptation. Many defense mechanisms were also transferred (category V), allowing for additional rapid protection against antimicrobials, perhaps associated with their soil niches or associated with increased capability to infect the human host. Overall, with the exception of the *M. smegmatis* group, horizontal acquisition of genes from non-mycobacterial species does not seem to have a large impact on the structure of mycobacterial genomes.

### Distribution of virulence genes across NTM groups

We next focused on a list of manually curated virulence factors retrieved from the literature^21^,^22^,^43^,^44^ belonging to five main classes: PE/PPE proteins, ESX export systems, Mce proteins, Sec-dependent secretion system, and Tat export system (Supplementary Table 9).

PE/PPE proteins are expressed on the cell surface and interact with the host’s immune system^45^. These gene families were initially studied because of their genetically hypervariable nature^46^, and they may play a role in evasion of host immune response, possibly via antigenic variation. It is known that these genes are unique to mycobacteria and are particularly abundant in pathogenic mycobacteria, such as *M. tuberculosis*. Their transport relies on the functionality of dedicated secretion pathways defined as ESX or type VII secretion system. Several studies have provided evidence for the involvement of one or more of these systems (especially ESX-1 and ESX-5) in pathogenesis and macrophage escape^47^. Here, we found that genes encoding PE/PPE proteins are almost exclusively present in *M. tuberculosis*, except for PE5, which is associated to the ESX-3 system and is present in most NTM species. The five ESX export systems are heterogeneously distributed across NTM complexes/groups: ESX-3 is conserved in all groups while ESX-1 seems to be specific for *M. tuberculosis. Mycobacterium abscessus* group possesses only the ESX-3 system.

**Figure 4:**
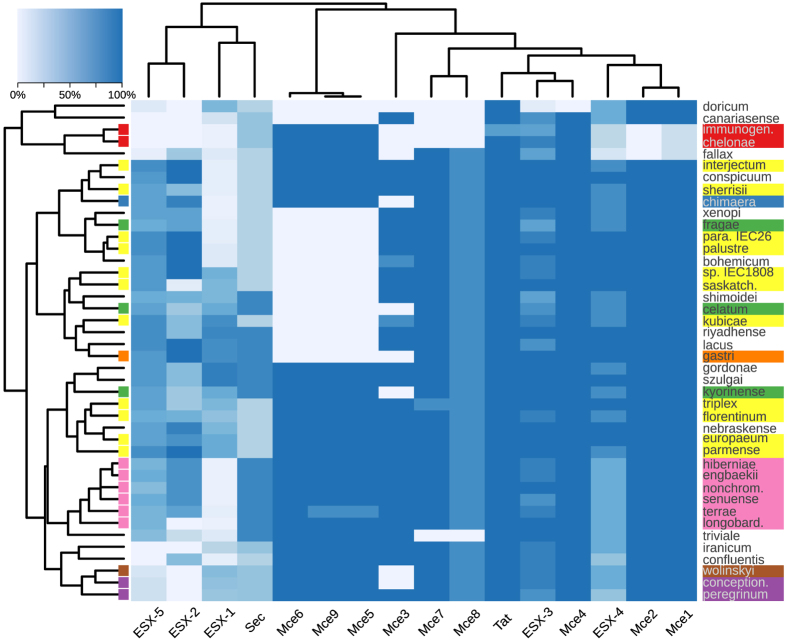
Distribution and abundance of virulence factors within the newly sequenced genomes. Cell intensity represents the fraction of genes in the corresponding gene family present in a given genome.

The Mce family proteins are involved in the invasion and persistence of mycobacteria in host macrophages and non-phagocytic mammalian cells^24^. The genes belonging to Mce5, Mce6, Mce7, Mce8 and Mce9 operons are absent in *M. tuberculosis* but are present in most NTM species, while Mce1, Mce2, Mce3 and Mce4 are present in *M. tuberculosis* and in many NTM species. In particular, Mce4 is highly conserved among all the groups, supporting observations from previous studies^24^. The distribution of Mce3, Mce5, Mce6 and Mce9 shows a significant association with NTM complexes/groups, except for the *M. simiae* complex that is also in this case very heterogeneous (p = 0.07). Moreover, Mce5, Mce6, and Mce9 are absent in one third of sequenced species and tend to co-occur when present while Mce1, Mce2 form a distinct group present in almost all species, with the exception of *M. abscessus* group and *M. fallax* (Fig. 4).

The Sec and Tat export systems occur in both Gram-negative and Gram-positive bacteria^48^ and act as the primary route for exporting proteins to the cytoplasmic membrane and beyond. SecA is a multifunctional component of the Sec export system and exported lipoproteins are known to be important for *M. tuberculosis* virulence^49^. Mycobacteria are naturally resistant to β-lactam antibiotics due to the production of β-lactamases secreted by the Tat export system^50^. In addition, *M. tuberculosis* Tat system plays a role in pathogenesis, as some of the known Tat-exported proteins of *M. tuberculosis* have a documented function in virulence^51^. Here we found that the Tat export system is ubiquitous in all the species sequenced in this study. Also the genes encoding SecA1, SecA2 and YajC proteins belonging to the Sec system are conserved in all the species, while the other components of the system are present only in some species but show a significant association with NTM group/complex (p ≤ 0.01).

A few genes, such as *secY*, that are coding for important functions involved in bacterial survival and host-bacterial interactions were absent in some species. However, the distribution of virulence genes in NTM species is consistent with the phylogenetic relationships based on the whole genome for the *M. terrae* complex, *M. abscessus* group and *M. fortuitum* complex; on the contrary members of the *M. celatum* group and *M. simiae* complex are splitted in three clusters each. The clustering of *M. nebraskense* and *M. bohemicum* based on the distribution of virulence factors corroborate the unexpected position of the *M. simiae* complex according to whole-genome phylogeny (Fig. 1) and also the functional profiles of *M shimoidei* and *M. celatum* are very similar.

### Genomic features of cell wall lipids strongly correlate with the NTM species complex

We also examined the distribution of genes encoding enzymes involved in mycolic acids (MA) and dimycocerosate esters (DIM) biosynthesis and assessed their possible association with complexes/groups, as well as with MA profiles detected by High-Performance Liquid Chromatography (HPLC)^52^. Proteins encoded by genes annotated from the genome of *Mycobacterium tuberculosis* H37Rv were used as a reference to identify orthologues in NTM species. This list of genes was retrieved from the literature^53^,^54^,^55^,^56^.

**Figure 5:**
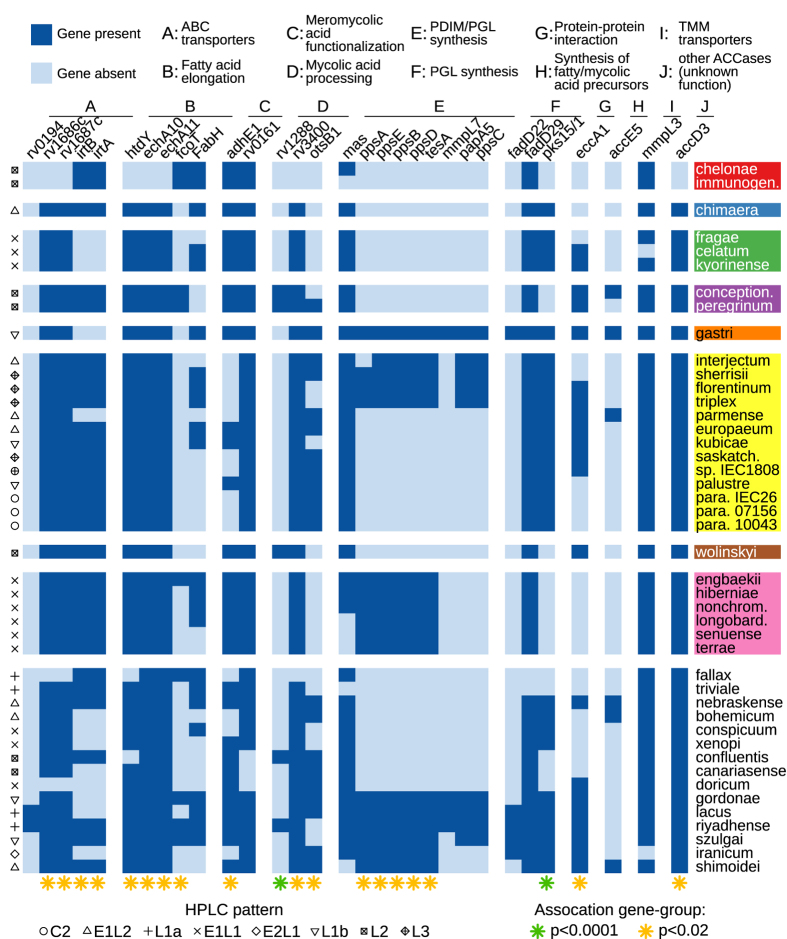
Heatmap representing the presence of genes driving the biosynthesis of mycolic acids and other key components of the cell envelope within the newly sequenced genomes. Genes are organized according to their role in MA or DIM biosynthesis pathways; species are grouped according to their assignment to complexes/groups. Only the genes that are absent from at least 2 of the genomes are shown (see Supplementary Table 10 for the complete list). On the left HPLC patterns of mycolic acids for each species are reported. Each HPLC pattern is identified by an acronym describing the number and the time of retention of peak clusters in the chromatogram as follows: two continuous sequences of peaks (C2), one early and two late clusters of peaks (E1L2), one late cluster of peaks (L1a, tuberculosis-like), one early and one late cluster of peaks (E1L1), two early and one late clusters of peaks (E2LI), one late cluster of peaks (L1b, kansasii-like), two late clusters of peaks (L2), three late clusters of peaks (L3).

Genes involved in MA biosynthesis were revealed to be highly conserved across NTM species (Supplementary Table 10), supporting their essential role in mycobacterial physiology. In contrast, the majority of the NTM species sequenced in this study lacked most DIM biosynthesis genes, which are known to be shared by only some of the slow growers, in particular the pathogenic mycobacteria: *M. tuberculosis* complex, *M. leprae, M. kansasii, M. marinum, M. ulcerans*, and *M. haemophilum*^56^. The *M. simiae* complex was found to contain species either with or without DIM biosynthesis genes, while the other complexes/groups had a more homogeneous distribution. We examined the association between MA and DIM biosynthesis genes with complexes/groups. A significant association was found for 19 genes (p ≤ 0.01). The strongest association was found for *pks15/1 (p* < 0.0001), a polyketide synthase responsible for the elongation of p-hydroxybenzoic acid (pHBA) with malonyl-CoA units, a key reaction for the formation of 17-(p-hydroxy-phenyl)-heptadecanoyl precursor of phenolic glycolipids (PGLs)^57^. Interestingly we did not find any significant association of *fabH* presence/absence with complex/groups (p = 0.48); the association became however significant (p = 0.006) once the four subgroups of *M. simiae* complex identified in our whole-genome phylogeny was considered separately (Fig. 1). Surprisingly very limited association was detected between presence/absence of MA biosynthesis genes and the type of HPLC profile. A significant association was found only for 5 genes (p ≤ 0.01) among the 19 studied. The distribution of MA and DIM biosynthesis enzymes corroborate the unexpected location of several species (*M. bohemicum* and *M. nebraskense*) revealed by the core genome phylogeny (Fig. 5).

## Conclusions

We applied here whole-genome shotgun sequencing to expand our knowledge on the genomic features of poorly characterized NTMs. Our genomic reconstructions of 41 NTM species, allowed for an almost doubling of the number of unique gene families occurring within the *Mycobacterium* genus. The analysis revealed an open pan-genome indicating that most of the functional diversity of mycobacteria remains to be characterized. Altogether, our work highlights the diversity of organisms in this genus and provides the first comprehensive whole genome characterization of NTMs.

Our phylogenetic analysis was based on a genome-wide alignment of hundreds of conserved genes which is recognized as one of the most reliable estimates to study the molecular evolution of organisms. On one hand we could confirm previously established relationships of NTM species based on sequencing of a single or few markers. In particular we found clearly distinct evolutionary pathways for slow and rapidly growing mycobacteria in agreement with the pre-NGS era phylogeny. On the other hand our analysis allowed the reclassification of misplaced species in particular in the *M. simiae* complex. Also the validity of the latter complex was not supported, being both paraphyletic with respect to *M. avium*, and composed of well-defined subgroups.

Importantly, the annotation of the new NTM genomes revealed that a vast majority (>60%) of predicted genes could not be assigned a specific function. The validity of most mycobacterial complexes and their functional specialization were highlighted by the high number of genes shared by members of a given complex and absent in the others. Although associating known gene functions of group-specific NTM with phenotypic traits is still challenging, our sequence-based gene clustering could lay the basis for further analysis aimed to identify markers genes at various taxonomic levels. These genes could have potential biomedical applications enabling the development of reliable diagnostic tools for the identification of a large number, if not all, of NTM species in clinical settings.

## Materials and Methods

We selected 47 Mycobacterium species representative of different members of the genus. Eleven rapidly growing and 36 slowly growing strains were included. Species whose genomes were already present in available databases were not included in our panel. With the aim of better understanding the phylogenetic correlations between the species we focused on those that, on the basis of conventional data, appeared to be members of complexes or groups. Among rapid growers, M. chelonae and M. immunogenum were selected as members of the M. abscessus complex; similarly M. peregrinum and M. conceptionense for the M. fortuitum complex and M. wolinskyi for the M. smegmatis group. Among slow growers the major targets of our investigation were the M. simiae complex (with nine species), the M. terrae complex (7 species), and the M. celatum group (3 species). Only one species of the M. avium complex was selected due to the presence of multiple genomes of such group in the databases. M. doricum was included to investigate thoroughly the ambiguous location of this slow grower within the phylogenetic branch of rapid growers; M. triviale to make clear its relatedness with the M. terrae complex. The other species were selected as representative either of potentially pathogenic or nonpathogenic organisms.

### Cultivation and DNA extraction

Strain cultivation was performed using liquid or solid media and incubation at 37 or 30 °C for enhancement of growth. For liquid cultures a fully automated system (BACTEC MGIT 960 Mycobacterial Detection System, BD Diagnostic Systems, Sparks, MD, USA) was used while Lowenstein-Jensen and Middlebrook 7H10 agar (BD Diagnostic Systems) were used as solid media.

Purified genomic DNA was obtained using a specific protocol for mycobacteria including enzymatic digestion, mechanical disruption of the cell wall and extraction with phenol/chloroform/isoamyl alcohol 25:24:1^58^. To confirm the strains identity *hsp65, rpoB* and 16S rRNA genes were partially sequenced by the Sanger method.

### Library preparation and high-throughput sequencing

Extracted DNA was quantified using Qubit 2.0 fluorometer (Invitrogen by ThermoFisher Scientific, Life Technologies Italia, Monza, Italy). Paired-end libraries were prepared from 1 ng of total bacterial DNA using Nextera XT DNA Sample Preparation kit and Nextera XT Index kit (Illumina Inc., San Diego, California, USA) according to manufacturer’s protocol. Library concentration and average fragment size were calculated by Qubit 2.0 fluorometer and Caliper LabChip GXI System (Perkin Elmer, Waltham, USA) respectively. Libraries were then normalized to 2 nM, pooled for multiplexing in equal volumes, and sequenced at 10 pM on the Illumina HiSeq 2000 platform (Illumina Inc., San Diego, California, USA) with 100 nt paired-end reads to achieve a coverage >100x per base.

### Genome assembly and annotation

The reads obtained from the sequencing phase were trimmed with trim galore (-q 0 –fastqc –nextera –stringency 5 –paired –retain_unpaired). The trimmed reads were assembled using the SPAdes assembler^29^ (ver. 3.5.0). This pipeline consists of three steps: read correction (corrections of sequencing errors based on other reads), read assembly (assembly of the reads and creation of contigs and scaffolds) and mismatch correction (correction of mismatches and short indels in the assembly).

We first verified that all the genomes had a consistent number of universal marker genes as defined by PhyloPhlAn^59^. With the exception of *M. leprae*^60^ which is known to be a species that underwent massive genomic reduction, the other genomes comprise an average 318 of the 400 universal markers and none of the genomes differed by more than 15 genes from this average (Supplementary Table 11). The assembled genomes were annotated with the Prokka pipeline^32^ (ver. 1.11) which integrates a set of tools specifically developed to annotate different genomic features. The quality of the annotated genomes were evaluated based on the work of Land *et al*.^61^. No genome was found to have a score lower than 0.82 (average 0.92 ± 0.04).

The eggNOG version 4 database (retrieved 21/04/15)^39^ was used to assign COG and NOG categories to all genomes. First, all proteins in the genomes were compared to the eggNOG database using the UBLAST algorithm implemented in USEARCH version 7.0.959^62^ with an e-value of 1e-30 and a bit-score cutoff of 70% of the top hit to ensure only close matches were retrieved and reduce the likelihood of spurious annotations. An eggNOG membership is assigned to each protein if 70% of the UBLAST hits belong to the same eggNOG member. Distinctions are then made between proteins with no UBLAST hit to any eggNOG sequence (no_hit), hits to a member that is not assigned an eggNOG code (none), and those without a 70% agreement (unassigned). Annotations are also clustered at the 25 higher COG functional category levels as per the eggNOG assignments.

The Pfam-A database (retrieved 20/05/15)^41^ was used to assign Pfam domain annotations to all proteins. Each protein set was compared to a hidden markov model version of Pfam-A using the hmmscan program of HMMER v3.1b2 (www.hmmr.org) using an e-value cut-off of 1e-3, producing a domain table for each sample. The statistical tests for the over- or under-representation of genes and gene families in groups of genomes have been performed using the chi-squared test.

The annotation pipeline (starting from the step of running Prokka) on the raw genomes was performed identically on the new genomes and on the already available ones to avoid potential inconsistencies in the downstream analysis.

### Pangenome and core genome reconstruction

An *ad-hoc* pipeline has been used to construct the pangenome. The pipeline starts by clustering all the genes in all genomes using vsearch version 1.0.5^63^ with a nucleotide percentage identity threshold of 80%. The clusters were then filtered based on their completeness in terms of prevalence in the considered strains (80%). The centroids (representatives) of the selected clusters according to vsearch were then used as query sequences for a BLAST search on the whole gene set (e-value: 1e-5, identity: 50%) discarding matches shorter than half of the closest reference sequence. The obtained clusters were then filtered to keep only the ones present in all strains to identify the genes from the core genome. The doubling of the total number of genes in the pangenome when adding the newly sequenced genomes to the set of already available genomes, has been validated by Roary^41^1.5 which found an increase in pangenome size of 1.9 times when clustering at 80% protein identity (from 88,391 to 167,536 gene families) and of 2.3 times when clustering at 95% protein identity (from 155,548 to 359,004 gene families). In addition, also the number of core genes identified by Roary (179 at 80% protein identity) is consistent, albeit smaller, than what we found here (243 core genes).

### Core gene and gene presence/absence phylogenetic reconstruction

The genes that are part of the core genome were then concatenated and aligned with MUSCLE^64^. The alignment was used as input for RAxML^65^ (ver. 8.0.9) with the GTRGAMMA model (with bootstrapping) to infer the core-genome phylogeny (Fig. 1). The gene presence/absence tree (Supplementary Figure 2) was built by converting the clusters to a binary matrix encoding the presence of each strain in the clusters and applying RAxML with the BINGAMMA model.

### Plasmid and phage detection

In order to detect potential plasmids and (pro)phage sequences within each genome, four separate approaches were used: homology, read depth and two circular contig detection methods. Since only homology-based methods would allow for differentiation of plasmid from phage, these two groups were combined in one analysis. Specifically, a contig that was found by any of the below four methods was considered a potential plasmid or phage.

#### BLAST to known plasmids and phages

BLASTn^66^, as implemented in blast + version 2.2.31^67^, was used to compare all contigs to the NCBI^68^ plasmid database (ftp.ncbi.nlm.nih.gov/genomes/Plasmids; retrieved 19/5/15) and Actinobacteriophage database (http://phagesdb.org; retrieved 19/5/15) with an e-value cutoff of 1e-10. A hit was retained only if the aligned segments covered at least 90% of the contig and 50% of the plasmid or phage sequence. This ensures high identities of partial matches are not retained and increase the likelihood of the genome contig being a whole plasmid or phage. For the phage database, this was done using both the sample genome contigs as the query and phage sequences as the database as well as vice versa.

#### Detection of circular contigs

In order to detect novel plasmids, genome contigs were analyzed to see if they were potentially circular. This occurs if the ends of the contigs had significant overlap with each other, suggesting circularity. The method by Jorgensen *et al*.^69^ was employed here. Briefly, contigs were cleaved in half and then reassembled using minimus2 from the AMOS package^70^. If the original ends of the contig are joined to form 1 new contig, this is likely circular in nature. Any such circular contigs over 1 kb in length were retained.

#### Read mapping overlap of contig ends

If a contig is circular in nature, it is likely that sequenced reads would overlap both ends of the linear representation of the contig. In order to test this, the paired-end information of our sequencing dataset was employed as described by Jorgensen *et al*.^69^. First, 500 bp were trimmed from both ends of each contig. BLASTn was then used to compare the raw read pairs to these contig ends with an e-value of 1e-10 and a 100% identity. If a read has a significant match to one contig end and its pair to the other, this is suggestive of a circular plasmid.

#### Potential plasmids based on read depth comparisons

Plasmid copy number is often much higher than that of the chromosome within each cell. This would likely refer to greater sequencing depth of contigs on plasmids compared to those on the chromosome. This was exploited as per Dhanani *et al*.^71^. All contigs found to contain a ribosomal gene were labelled as the chromosome set. Any contig found to have an average read depth 10 times greater than the average depth of all contigs in the chromosome set was labelled as a potential plasmid.

### Detection of lateral gene transfer events

In order to determine which genes were potentially acquired through LGT a homology/taxonomy approach was employed using HGTector v0.1.8^72^. This analysis pipeline compares protein sequences (labelled the self set) to a database of potential donors and assesses whether there is a higher abundance of closely related sequences in the distal set than the close set. Here, each sample was labelled with the taxonomy of the closest known Mycobacterium species (labelled the self set) with the genus Mycobacterium as the close set and all other species as the distal set. This method allows for multiple Mycobacterium species to contain the potentially transferred gene but still be detected as an LGT event. In order to do this, the full proteome of each sample was compared to the NCBI NR database (retrieved 30/03/15) using USEARCH with an e-value cutoff of 1e-30 and an 80% identity score. These hit datasets were then converted to.bla files for input to HGTector using custom python scripts. The taxonomy of each hit was determined using the HGTector taxonomer.pl script and the NCBI taxonomy (retrieved 30/03/15)^68^. The e-value and percent identity values for HGTector were set as 1e-30 and 80% respectively.

Orthologous gene replacement and recombination events between Mycobacterial species was searched for using Gubbins^73^. The core genes concatenated alignment was used as input and any recombination events that were longer than 1 kb were marked as significant.

Data Avaliability. The genomes are available at NCBI under project ID PRJNA299467 and PRJNA308282.

## Additional Information

**How to cite this article:** Fedrizzi, T. *et al*. Genomic characterization of Nontuberculous Mycobacteria. *Sci. Rep.* **7**, 45258; doi: 10.1038/srep45258 (2017).

**Publisher's note:** Springer Nature remains neutral with regard to jurisdictional claims in published maps and institutional affiliations.

## Supplementary Material

Supplementary Materials

Supplementary Table 10

Supplementary Table 9

Supplementary Table 11

## Figures and Tables

**Table 1:** Summary table for the high quality draft assemblies obtained.

Complexes and groups	# already sequenced species	# newly sequenced species	N50 (kb)	N90 (kb)	GC (%)	# ORFs	Quality score (%)	# contigs	Length (mb)
*M. abscessus*	1	2	605 ± 470	214 ± 170	64.1 ± 0.3	5253 ± 402	96.6 ± 0.7	22 ± 18	5.3 ± 0.4
*M. avium*	4	1	115	26	67.7	5777	91.3	108	6.1
*M. celatum*	0	3	173 ± 106	53 ± 35	66.6 ± 0.5	4968 ± 600	93.2 ± 1.7	91 ± 78	5.0 ± 0.5
*M. fortuitum*	2	2	261 ± 84	84 ± 23	66.3 ± 0.1	6593 ± 410	94.3 ± 1.1	60 ± 25	6.8 ± 0.4
*M. kansasii*	1	1	85	23	66.2	5333	92.3	154	5.8
*M. simiae*	1	11 (1)	211 ± 113	53 ± 26	68.1 ± 1.1	5620 ± 217	91.9 ± 4.1	81 ± 36	5.9 ± 0.2
*M. smegmatis*	1	1	481	168	66.4	7392	93.9	36	7.5
*M. terrae*	1	6	153 ± 15	51 ± 14	68.3 ± 0.4	4283 ± 209	91.6 ± 3.9	63 ± 18	4.5 ± 0.2
Other species	25 (8)	15	131 ± 64	34 ± 17	67.4 ± 1.7	5265 ± 1119	92.0 ± 3.2	125 ± 71	5.6 ± 1.2

We report indicators of reconstruction quality (N50, N90, number of contigs), intrinsic sequence characteristics (percentage GC content, total genome length), gene characteristics (number of ORFs), overall quality and completeness (quality score computed as described elsewhere^61^). The numbers in parenthesis represent how many strains are part of a complex but do not have an assigned species. See Supplementary Table 1 for the characteristics of each sequenced genome.

**Table 2:** Detection of mobile elements in *Mycobacterium* genomes.

Complex	No. species with plasmid/phage	Mobile element count	Average mobile elements per species
M. avium	1	3	3
M. kansasii	1	2	2
M. simiae	3	6	2
no complex/group	4	15	3.75

The number of plasmid or phage detected in each species is summarised at the complex level. Detailed are the number of species per complex with a plasmid/phage, the total count of plasmid/phage per complex and the average per species within that complex.

**Table 3:** Summary of estimated amount of lateral gene transfer per complex.

Complex	Minimum (%)	Average (%)	Maximum (%)
*M. abscessus*	0.84	0.91	0.97
*M. avium*	0.32	0.32	0.32
*M. celatum*	0.04	0.16	0.22
*M. fortuitum*	0.5	0.63	0.75
*M. kansasii*	0.04	0.04	0.04
*M. simiae*	0.11	0.47	1.78
*M. smegmatis*	1.9	1.9	1.9
*M. terrae*	0.07	0.16	0.24
Other species	0.07	0.33	0.78

The contribution of LGT to genetic material per sequenced genome was calculated. Outlined in the table are the minimum, average and maximum percent derived from LGT per genome within each complex.
