# Supplementary material for: Genomic characterization of Nontuberculous Mycobacteria
Source: Sci Rep. 2017 Mar 27;7:45258. doi: 10.1038/srep45258 (PMC5366915; doi:10.1038/srep45258)
Supplement: Supplementary Materials [file srep45258-s1.pdf]

Genomic characterization of Nontuberculous Mycobacteria

Tarcisio Fedrizzi [*,1], *Conor J. Meehan* [*,2], *Antonella Grottola* [3], *Elisabetta Giacobazzi* [1], *Giulia Fregni Serpini* [3], *Sara Tagliazucchi* [3], *Anna Fabio* [3], *Clotilde Bettua* [1], *Roberto Bertorelli* [4], *Veronica De Sanctis* [4], *Fabio Rumpianesi* [3], *Monica Pecorari* [3], *Olivier Jousson* [1], *Enrico Tortoli* [+,5], *Nicola Segata* [+,1]

1. Centre for Integrative Biology, University of Trento, Trento, Italy
2. Mycobacteriology unit, Department of Biomedical Science, Institute of Tropical Medicine, Antwerp, Belgium
3. Microbiology and Virology Unit, University Hospital Polyclinic, Modena, Italy
4. NGS Facility, Laboratory of Biomolecular Sequence and Structure Analysis for Health, Centre for Integrative Biology, University of Trento, Italy
5. Emerging Bacterial Pathogens Unit, IRCCS San Raffaele Scientific Institute, Milano, Italy

*** These authors contributed equally**

+ Corresponding authors: e.tortoli@libero.it; nicola.segata@unitn.it

Supplementary Figures and Tables

Supplementary Figures

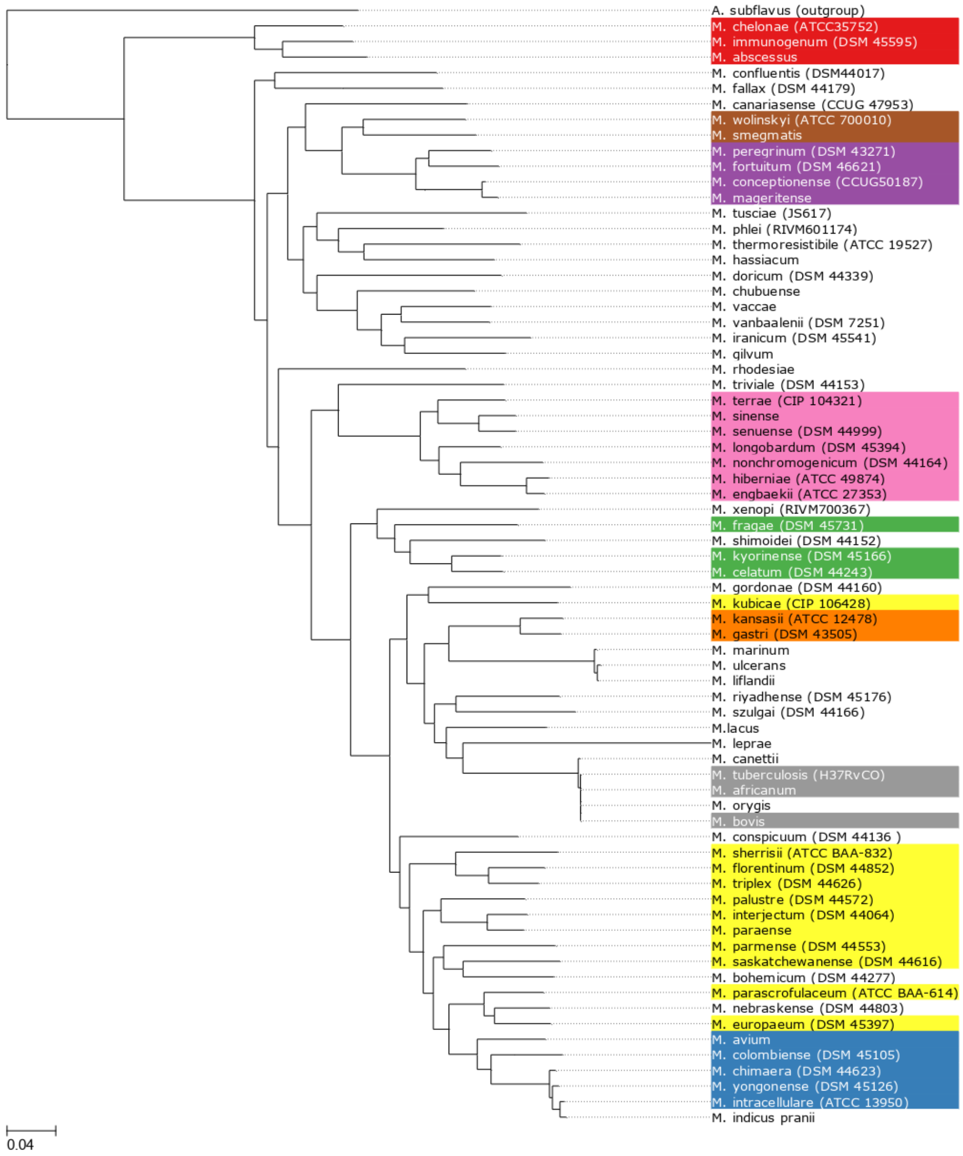

Supplementary Figure 1: Reduced tree containing a single strain per species

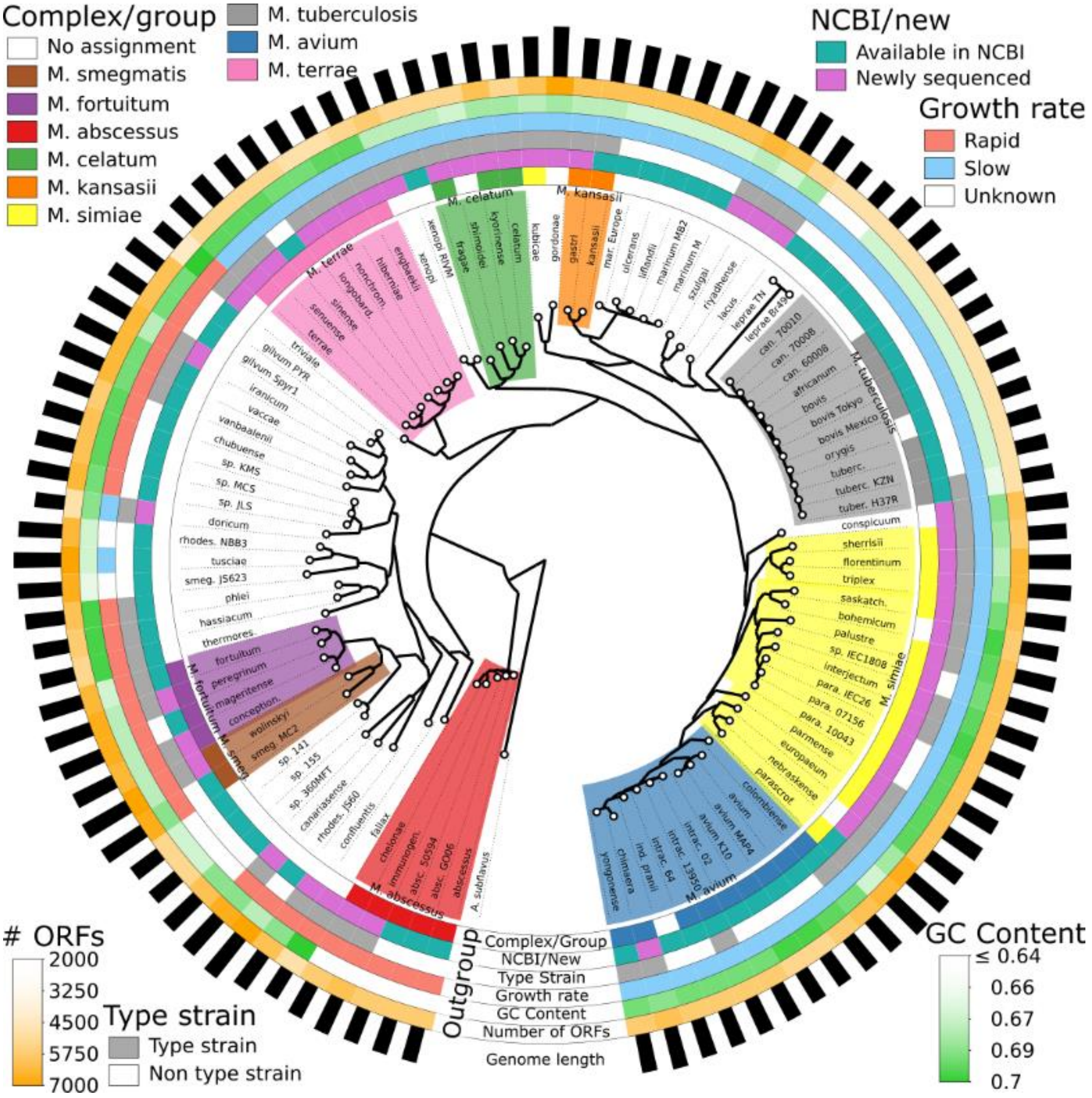

Supplementary Figure 2: presence-absence hierarchical clustering of the Mycobacteria genus. The tree is built based on the presence-absence of genes within the genus with the Maximum-likelihood inference approach implemented in RAxML (**see Methods**) and displayed using GraPhlAn [28]. Colored shades and external circles are the same as the one described in **Figure 1**

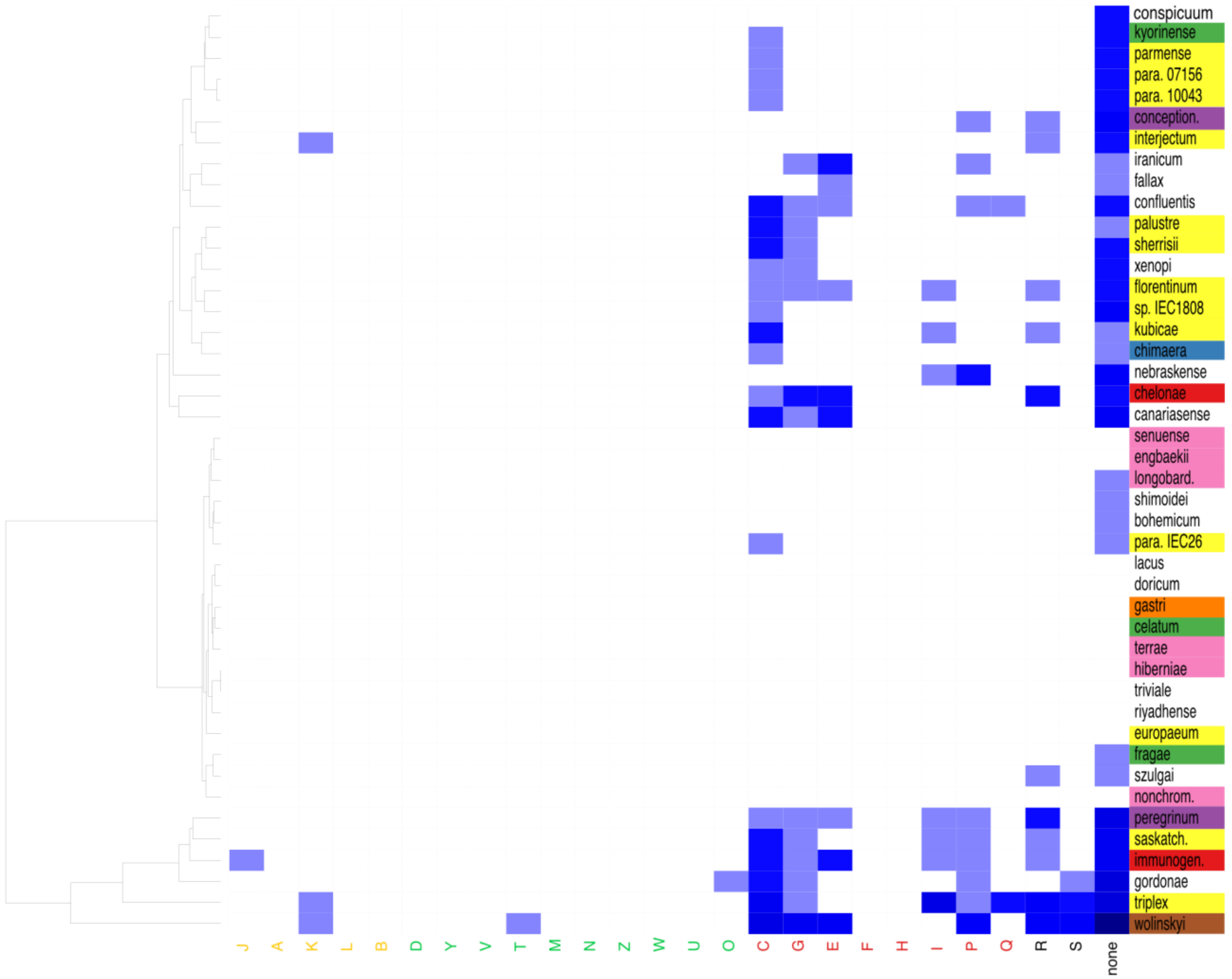

Supplementary Figure 3: Distribution of COG categories acquired through LGT in each genome. The number of genes in each genome derived from LGT was calculated and displayed as intensities in a heatmap (white=0; dark blue=43). Genes are separated into the 4 high level COG categories: information storage and processing (yellow), cellular processing and signalling (green), metabolism (red) and poorly characterized (black). Strains are coloured according to their complex.

Supplementary Tables

Supplementary Table 1: The characteristics of the reconstructed genomes.

species	Complex/group	# raw reads	# QC'd reads	coverage	#contigs	N50	N75	N90	Average contig length	Quality scores	Average read length	Genome length	GC content	# ORFs
M. fallax	no complex/group	9855260	5001001	110	84	131191	59487	26883	50387	93.40%	93.1	4232505	70.31%	4060
M. bohemicum	no complex/group	11623716	5962905	103	52	220473	107715	53813	104241	95.35%	93.2	5420516	69.18%	5050
M. canariasense	no complex/group	12165286	6418709	88	113	121943	59409	39130	59784	93.95%	92.2	6755573	67.59%	6900
M. nebraskense	no complex/group	12351342	6506621	90	189	86703	49493	20470	35594	87.05%	93.4	6727177	66.62%	6412
M. doricum	no complex/group	11298294	5356971	127	75	123422	74911	35014	52708	91.05%	93.7	3953129	67.55%	3876
M. celatum	M. celatum	18163524	7781615	155	57	162031	123999	55775	82819	94.85%	93.8	4720680	66.96%	4644
M. gastri	M. kansasii	12933772	6505634	105	154	85262	48630	23371	37771	92.30%	93.5	5816768	66.20%	5333
M. senuense	M. terrae	11600502	5509673	114	54	153375	93870	57747	83975	94.88%	94.1	4534628	68.69%	4290
M. gordonae	no complex/group	21261044	9600117	120	260	62676	36083	15693	29237	91.15%	94.7	7601632	66.59%	6985
M. nonchromogenicum	M. terrae	20339488	8165690	173	91	138127	61279	25752	49070	85.80%	94.4	4465329	67.77%	4163
M. peregrinum	M. fortuitum	15033344	7766812	104	78	201665	102455	67598	90387	95.05%	94.5	7050152	66.26%	6883
M. paraense FI 10043	M. simiae	9418064	5380537	86	51	236783	149252	58309	113846	95.53%	92.5	5806129	68.98%	5594
M. shimoidei	no complex/group	20455436	8208873	164	71	128027	75340	47758	66391	94.28%	94.3	4713740	65.77%	4580
M. sp. IEC 1808	M. simiae	9971110	5558325	85	72	141910	78715	38278	84881	92.40%	93.2	6111456	68.23%	5873
M. szulgai	no complex/group	18967982	8629301	122	178	95710	59984	24062	37487	92.25%	94.5	6672659	65.85%	5937
M. paraense IEC26	M. simiae	12491844	6621577	108	51	271458	110065	63427	110187	92.95%	91.9	5619528	69.26%	5369
M. triplex	M. simiae	12844984	6660124	98	48	258006	138580	79033	132632	93.28%	93.5	6366331	66.60%	6047
M. triviale	no complex/group	21031866	8648390	223	57	127871	74656	38575	63001	94.13%	92.6	3591083	70.28%	3488
M. xenopi	no complex/group	11534220	5499907	104	146	71256	37642	21207	33790	84.33%	93.4	4933400	65.90%	4899
M. chelonae	M. abscessus	20487374	8091410	153	9	936739	595964	334843	558196	97.10%	95.3	5023765	63.92%	4969
M. chimaera	M. avium	11752606	6180763	95	108	115409	87017	25936	56360	91.25%	93.2	6086879	67.70%	5777
M. confluentis	no complex/group	9299072	5122979	83	57	244628	119370	77849	102486	95.33%	94.9	5841691	67.51%	5569
M. paraense FI 07156	M. simiae	9714418	5448598	87	46	311494	205432	89332	126206	95.70%	93.0	5805490	68.98%	5586
M. conspicuum	no complex/group	9644810	5292267	81	92	221692	65212	34238	67398	94.30%	94.7	6200614	67.37%	5768
M. conceptionense	M. fortuitum	18678712	8590387	126	42	320022	189176	100036	154019	93.50%	94.8	6468817	66.44%	6303
M. florentinum	M. simiae	12541726	6767439	101	34	518602	216401	105827	181718	96.23%	92.4	6178409	66.38%	5892
M. fragae	M. celatum	12679874	6063590	119	35	284076	158038	86395	135173	93.33%	93.2	4731047	66.01%	4599
M. europaeum	M. simiae	10276102	5537929	92	71	163313	90679	53844	79305	89.73%	93.5	5630674	68.51%	5330
M. engbaekii	M. terrae	20888940	8427266	175	79	132590	87302	43406	57233	88.83%	93.9	4521435	68.55%	4207
M. iranicum	no complex/group	17141494	8024244	120	82	240675	99791	50588	77264	94.68%	94.6	6335651	66.10%	6156
M. kyorinense	M. celatum	11767964	6098808	101	180	71978	41315	16621	31148	91.45%	92.8	5606671	66.75%	5660
M. longobardum	M. terrae	16524904	7426178	145	59	159248	111035	57523	81564	94.80%	93.8	4812247	67.92%	4686
M. palustre	M. simiae	10145826	5542159	86	158	85200	45038	21909	38213	82.35%	93.8	6037683	68.50%	5778
M. parmense	M. simiae	11417560	5773564	93	91	177034	68562	39759	64744	94.18%	94.7	5891740	68.39%	5540
M. riyadhense	no complex/group	13861214	6890514	103	263	56910	33434	12433	23840	87.63%	93.6	6269898	65.28%	5780
M. lacus	no complex/group	22915038	8930803	171	187	55464	31204	15430	26231	90.63%	94.1	4905288	66.90%	4608
M. saskatchewanense	M. simiae	6361074	3747442	61	87	152462	75612	33667	68172	89.33%	96.0	5930969	68.28%	5597
M. terrae	M. terrae	20177906	8569873	177	47	169342	128105	57049	96273	94.95%	93.3	4524815	68.44%	4257
M. wolinskyi	M. smegmatis	19192038	9362819	117	36	481307	266081	168281	208802	93.90%	94.2	7516859	66.42%	7392
M. kubicae	M. simiae	12101666	6208654	100	116	134284	74830	30432	50281	95.88%	93.5	5832620	66.01%	5557
M. sherrisii	M. simiae	20891914	8857194	150	128	125930	54633	22784	43454	85.35%	94.1	5562166	67.02%	5308
M. interjectum	M. simiae	16637160	7960981	126	103	103926	66066	37873	57319	91.33%	93.4	5903836	68.56%	5666
M. immunogenum	M. abscessus	12957922	6315698	107	35	272673	214817	93749	158463	96.05%	94.3	5546219	64.28%	5537
M. hiberniae	M. terrae	11344388	5751413	122	48	165395	117472	65728	90462	90.05%	92.4	4342192	68.53%	4093

Supplementary Table 2: Abbreviations used in the figures.

Complete name	Abbreviation	RefSeq assembly accession
M. abscessus	abscessus	GCF_000333695
M. abscessus subsp bolletii 50594	absc. 50594	GCF_000445035
M. abscessus subsp bolletii str GO 06	absc. GO06	GCF_000277775
M. africanum GM041182	africanum	GCF_000253355
M. avium 104	avium	GCF_000014985
M. avium subsp paratuberculosis K 10	avium K10	GCF_000007865
M. avium subsp paratuberculosis MAP4	avium MAP4	GCF_000390085
M. bovis AF2122 97	bovis	GCF_000195835
M. bovis BCG str Mexico	bovis Mexico	GCF_000234725
M. bovis BCG str Tokyo 172	bovis Tokyo	GCF_000010685
M. canettii CIPT 140060008	can. 60008	GCF_000328805
M. canettii CIPT 140070008	can. 70008	GCF_000328845
M. canettii CIPT 140070010	can. 70010	GCF_000328785
M. chubuense NBB4	chubuense	GCF_000266905
M. colombiense CECT 3035	colombiense	GCF_000222105
M. fortuitum subsp fortuitum DSM 46621	fortuitum	GCF_000295855
M. gilvum PYR GCK	gilvum PYR	GCF_000016365
M. gilvum Spyr1	gilvum Spyr1	GCF_000184435
M. hassiacum DSM 44199	hassiacum	GCF_000300375
M. indicus pranii MTCC 9506	ind. pranii	GCF_000298095
M. intracellulare ATCC 13950	intrac. 13950	GCF_000277125
M. intracellulare MOTT 02	intrac. 02	GCF_000277145
M. intracellulare MOTT 64	intrac. 64	GCF_000276825
M. kansasii ATCC 12478	kansasii	GCF_000157895
M. leprae Br4923	leprae Br49	GCF_000026685
M. leprae TN	leprae TN	GCF_000195855
M. liflandii 128FXT	liflandii	GCF_000026445
M. mageritense JR2009	mageritense	GCF_000233935
M. marinum M	marinum M	GCF_000018345
M. marinum MB2	marinum MB2	GCF_000419335
M. marinum str Europe	mar. Europe	GCF_000419315
M. orygis 112400015	orygis	GCF_000353205
M. parascrofulaceum ATCC BAA 614	parascrof.	GCF_000164135
M. phlei RIVM601174	phlei	GCF_000257725
M. rhodesiae JS60	rhodes. JS60	GCF_000230935
M. rhodesiae NBB3	rhodes. NBB3	GCF_000230895
M. smegmatis JS623	smeg. JS623	GCF_000328565
M. smegmatis str MC2 155	smeg. MC2	GCF_000015005
M. sp 141	sp. 141	GCF_000382405
M. sp 155	sp. 155	GCF_000373905
M. sp 360MFTsu5 1	sp. 360MFT	GCF_000383495
M. sinense	sinense	GCF_000214155
M. sp JLS	sp. JLS	GCF_000016005
M. sp KMS	sp. KMS	GCF_000015405
M. sp MCS	sp. MCS	GCF_000014165
M. *thermoresistibile* ATCC 19527	thermores.	GCF_000234585
M. tuberculosis	tuberc.	GCF_000331445
M. tuberculosis H37RvCO	tuber. H37R	GCF_000263135
M. tuberculosis KZN 605	tuberc. KZN	GCF_000154605
M. tusciae JS617	tusciae	GCF_000243415
M. ulcerans Agy99	ulcerans	GCF_000013925
M. vaccae ATCC 25954	vaccae	GCF_000295825
M. vanbaalenii PYR 1	vanbaalenii	GCF_000015305
M. xenopi RIVM700367	xenopi RIVM	GCF_000257745
M. yongonense 05 1390	yongonense	GCF_000418535
M. fallax	fallax	(this study)
M. bohemicum	bohemicum	(this study)
M. canariasense	canariasense	(this study)
M. nebraskense	nebraskense	(this study)
M. doricum	doricum	(this study)

M. celatum	celatum	(this study)
M. gastri	gastri	(this study)
M. senuense	senuense	(this study)
M.gordonae	gordonae	(this study)
M. nonchromogenicum	nonchrom.	(this study)
M. peregrinum	peregrinum	(this study)
M. paraense FI 10043	para. 10043	(this study)
M. shimoidei	shimoidei	(this study)
M. sp. IEC 1808	sp. IEC1808	(this study)
M. szulgai	szulgai	(this study)
M. paraense IEC26	para. IEC26	(this study)
M. triplex	triplex	(this study)
M. triviale	triviale	(this study)
M. xenopi	xenopi	(this study)
M. chelonae	chelonae	(this study)
M. chimaera	chimaera	(this study)
M. confluentis	confluentis	(this study)
M. paraense FI 07156	para. 07156	(this study)
M. conspicuum	conspicuum	(this study)
M. conceptionense	conception.	(this study)
M. florentinum	florentinum	(this study)
M. fragae	fragae	(this study)
M. europaeum	europaeum	(this study)
M. engbaekii	engbaekii	(this study)
M. iranicum	iranicum	(this study)
M. kyorinense	kyorinense	(this study)
M. longobardum	longobard.	(this study)
M. palustre	palustre	(this study)
M. parmense	parmense	(this study)
M. riyadhense	riyadhense	(this study)
M. lacus	lacus	(this study)
M. saskatchewanense	saskatch.	(this study)
M. terrae	terrae	(this study)
M. wolinskyi	wolinskyi	(this study)
M. kubicae	kubicae	(this study)
M. sherrisii	sherrisii	(this study)
M. interjectum	interjectum	(this study)
M. immunogenum	immunogen.	(this study)
M. hiberniae	hiberniae	(this study)
A. subflavus DQS3 9A1	A. subflavus	GCF_000214175

Supplementary Table 3: Average genome percentage of each COG category within the mycobacterial complexes.

Category	Abscessus	Avium	Celatum	Fortuitum	Kansasii	Simiae	Smegmatis	Terrae	No Complex	Avg
J	1.90	1.58	1.75	1.48	1.70	1.59	1.38	2.11	1.72	1.69
A	0.00	0.00	0.00	0.00	0.00	0.00	0.00	0.00	0.02	0.00
K	5.49	3.89	4.15	5.86	3.58	4.29	6.35	4.50	4.04	4.68
L	2.01	2.64	2.14	1.98	2.59	1.89	1.65	2.21	2.30	2.16
D	0.40	0.39	0.34	0.30	0.38	0.30	0.30	0.41	0.35	0.35
V	0.58	0.63	0.58	0.67	0.97	0.58	0.56	0.72	0.69	0.67
T	1.24	1.23	1.29	1.19	1.51	1.57	1.38	1.23	1.48	1.35
M	1.81	1.79	2.04	1.72	2.12	1.95	1.75	2.07	1.91	1.91
N	0.10	0.00	0.00	0.00	0.10	0.02	0.00	0.06	0.04	0.03
U	0.28	0.21	0.19	0.20	0.23	0.20	0.18	0.26	0.23	0.22
O	1.40	1.28	1.37	1.25	1.51	1.31	1.11	1.50	1.45	1.35
C	3.37	3.50	3.43	3.43	3.39	3.76	4.00	3.42	3.38	3.52
G	2.02	1.68	1.97	2.00	2.04	2.05	3.09	2.19	2.15	2.13
E	3.45	2.12	2.38	3.01	2.42	2.43	3.92	2.48	2.61	2.76
F	0.93	0.65	0.75	0.75	0.82	0.64	0.82	0.85	0.78	0.78
H	1.61	1.23	1.49	1.27	1.47	1.39	1.31	1.73	1.53	1.45
I	3.67	4.38	4.32	3.99	3.77	4.56	3.56	5.23	4.14	4.18
P	3.07	2.77	2.75	2.92	2.36	2.73	3.61	2.68	2.66	2.84
Q	2.52	2.99	2.60	2.33	2.84	2.90	2.06	2.58	2.41	2.58
R	7.50	6.27	6.64	7.12	5.78	6.83	7.06	6.75	6.19	6.68
S	15.66	12.87	13.28	13.12	13.31	12.97	12.72	14.14	12.76	13.42
Combinations	1.17	1.24	1.24	1.06	0.90	13.11	1.15	1.00	15.76	4.07
None assigned	25.48	34.49	29.65	28.09	33.64	31.63	26.77	32.00	30.43	30.24
No eggNOG homolog	14.34	12.19	15.66	16.26	12.58	1.30	15.28	9.90	0.98	10.94

Supplementary Table 4: Distribution of COG categories for genes specific to each complex. The total number of genes specific to a complex is shown along with the percentage of these genes belonging to each COG category.

Category	Abscessus	Avium	Celatum	Fortuitum	Kansasii	Simiae	Smegmatis	Terrae	Avg
# genes	731	51	173	428	140	995	409	431	419.75
J	0.82	0.00	0.00	0.23	0.00	0.30	0.24	0.46	0.26
A	0.00	0.00	0.00	0.00	0.00	0.00	0.00	0.00	0.00
K	11.08	15.69	10.98	16.59	5.71	8.04	15.40	10.67	11.77
L	1.37	0.00	4.62	3.97	8.57	0.60	0.73	0.70	2.57
D	0.27	0.00	0.00	0.00	0.71	0.00	0.00	0.46	0.18
V	1.23	0.00	0.00	0.93	1.43	0.50	0.24	0.93	0.66
T	1.78	1.96	1.73	0.70	2.86	5.33	3.42	2.09	2.48
M	1.64	1.96	1.73	0.70	2.86	2.81	1.71	1.16	1.82
N	0.00	0.00	0.00	0.00	0.00	0.00	0.00	0.00	0.00
U	0.41	0.00	0.00	0.23	0.00	0.00	0.00	0.00	0.08
O	1.09	0.00	0.58	0.93	2.86	0.90	0.98	0.70	1.01
C	1.64	0.00	2.31	0.47	0.00	6.03	1.71	2.09	1.78
G	3.28	1.96	1.73	3.97	3.57	5.23	9.78	5.80	4.42
E	4.38	1.96	3.47	2.34	0.71	1.91	6.36	2.09	2.90
F	0.55	0.00	1.16	1.40	0.00	0.00	0.00	0.46	0.45
H	0.96	0.00	0.00	0.70	2.86	0.00	0.73	1.62	0.86
I	3.01	1.96	2.31	1.17	3.57	1.81	0.98	2.55	2.17
P	3.01	1.96	1.16	3.04	2.14	3.92	5.38	2.55	2.89
Q	1.23	0.00	1.73	3.04	1.43	1.71	1.47	5.57	2.02
R	12.72	17.65	20.23	19.16	14.29	12.76	16.63	10.90	15.54
S	49.52	54.90	46.24	40.42	46.43	48.14	34.23	49.19	46.13

Supplementary Table 5: Complex specific clusters of genes. Genes found to be present in one complex and absent from others were clustered to find 5 or more such genes that were co-localised in the genomes. Three such clusters were found in the *M. abscessus* complex and one in the *M. fortuitum* complex.

***M. abscessus* cluster 1**
Putative enoyl-CoA hydratase/isomerase
Acetyl-CoA acetyltransferase
Nucleic-acid-binding protein containing a Zn-ribbon
Nucleic-acid-binding protein containing a Zn-ribbon
Hypothetical protein
Hypothetical protein
Hypothetical protein
Hypothetical protein
***M. abscessus* cluster 2**
Hypothetical protein
Hypothetical protein
Daunorubicin resistance ABC transporter ATP-binding subunit DrrA
ABC transporter transmembrane protein
Acyl carrier protein
***M. abscessus* cluster 3**
Glycosyl transferase
Glycosyl transferase
MOP superfamily O-antigen transporter
Hypothetical protein
Glycosyl transferase
Hypothetical protein

***M. fortuitum* cluster 1**
Putative salicylate hydroxylase
Hypothetical protein
2-hydroxy-3-carboxy-6-oxo-7-methylocta-2, 4-dienoate decarboxylase
Sldehyde oxidase and xanthine dehydrogenase
2Fe-2S iron-sulfur cluster binding domain-containing protein

Supplementary Table 6: Predicted mobile elements in mycobacterial genomes. Contigs of genomes predicted to be mobile elements are outlined here. The method by which they were predicted as mobile elements is listed, along with those which contained either primarily transposable elements (TE) or had phage keywords in the protein annotations.

Contig name	Method	Phage/TE keywords
M. canariasense		
NODE_100	Read depth	
NODE_101	Read depth	Phage
NODE_105	Read depth	
NODE_106	Read depth	
NODE_108	Read depth	
NODE_112	Read depth	Phage
NODE_116	Read depth	
NODE_70	Read depth	
NODE_78	Read depth	Phage
NODE_84	Read depth	Phage
NODE_93	Read depth	
M. chimaera		
NODE_39	Homology (plasmid)	Phage
NODE_43	Homology (plasmid)	Phage
NODE_75	Homology (plasmid)	
M. interjectum		
NODE_93	Read depth	TE
M. gastri		
NODE_123	Read depth	TE
NODE_138	Read depth	TE
M. lacus		
NODE_152	Read depth	TE
NODE_168	Read depth	TE
M. nebraskense		
NODE_185	Read depth	TE
M. palustre		
NODE_146	Read depth	
NODE_147	Read depth	
NODE_149	Read depth	TE
NODE_159	Read depth	
M. riyadhense		
NODE_264	Read depth	TE
M. saskatchewanense		
NODE_82	Read depth	TE

Supplementary Table 7. Number and percentage of transposases in each strain.

	Transposase count	% of genome	Total count
M. fallax	36	0.91	3954
M. nebraskense	114	1.81	6314
M. bohemicum	23	0.47	4892
M. doricum	46	1.21	3809
M. canariasense	36	0.53	6800
M. celatum	13	0.29	4557
M. gastri	58	1.11	5246
M. senuense	21	0.5	4213
M. gordonae	96	1.39	6909
M. nonchrom	28	0.68	4097
M. peregrinum	36	0.53	6797
M. paraense	6	0.11	5516
M. IEC 10745	19	0.33	5703
M. shimoidei	22	0.49	4508
M. szulgai	15	0.26	5848
M. para. 1392	7	0.13	5290
M. triplex	20	0.34	5967
M. para. 11978	8	0.15	5433
M. triviale	19	0.56	3405
M. xenopi	60	1.25	4813
M. chelonae	6	0.12	4898
M. chimaera	71	1.24	5711
M. confluentis	10	0.18	5494
M. para. 07156	6	0.11	5506
M. conspicuum	16	0.28	5677
M. conception	25	0.4	6227
M. florentinum	10	0.17	5818
M. fragae	10	0.22	4534
M. europaeum	27	0.51	5262
M. engbaekii	34	0.82	4136
M. iranicum	32	0.53	6084
M. kyorinense	68	1.22	5576
M. longobard	26	0.56	4607
M. palustre	16	0.28	5649
M. parmense	45	0.82	5469
M. riyadhense	73	1.28	5688
M. lacus	41	0.91	4525

M. saskatch	36	0.65	5525
M. terrae	14	0.34	4179
M. wolinskyi	33	0.45	7322
M. kubicae	94	1.71	5489
M. sherrisii	61	1.17	5221
M. interjectum	13	0.23	5589
M. immunogen	15	0.28	5453
M. hiberniae	9	0.22	4026

Supplementary Table 8: Lateral gene transfer in each mycobacterial genome. The number of genes and percentage of protein coding genes acquired through LGT is outlined in the table.

**Strain**	**LGT genes**	**% of genome from LGT**
M. bohemicum	35	0.55
M. canariasense	6	0.12
M. celatum	2	0.04
M. chelonae	41	0.84
M. chimaera	18	0.32
M. conceptionense	31	0.5
M. confluentis	31	0.56
M. conspicuum	15	0.26
M. doricum	38	0.56
M. engbaekii	5	0.12
M. europaeum	6	0.11
M. fallax	13	0.33
M. florentinum	28	0.48
M. fragae	10	0.22
M. gastri	2	0.04
M. gordonae	54	0.78
M. hiberniae	3	0.07
M. immunogenum	53	0.97
M. interjectum	25	0.45
M. iranicum	23	0.38
M. kubicae	24	0.44
M. kyorinense	12	0.22
M. lacus	5	0.11
M. longobardum	11	0.24
M. nebraskense	5	0.13
M. nonchromogenicum	10	0.24
M. palustre	24	0.42
M. paraense FI 07156	16	0.29
M. paraense FI 10043	15	0.27
M. paraense IEC26	7	0.13
M. parmense	19	0.35
M. peregrinum	51	0.75
M. riyadhense	4	0.07
M. saskatchewanense	42	0.76
M. senuense	8	0.19
M. sherrisii	22	0.42
M. shimoidei	25	0.44
M. sp. IEC1808	7	0.16
M. szulgai	10	0.17
M. terrae	4	0.1
M. triplex	106	1.78
M. triviale	3	0.09
M. wolinskyi	139	1.9
M. xenopi	20	0.42

Supplementary Table 9. Distribution of genes encoding for PE/PPE proteins, ESX export systems, Mce proteins, Sec-dependent secretion system, and Tat export system**.** (See attached Excel table).

Supplementary Table 10. Distribution of genes encoding enzymes involved in mycolic acids (MA) and dimycocerosate esters (DIM) biosynthesis in the newly sequenced genomes. (See attached Excel table)

Supplementary Table 11. Number of PhyloPhlAn universal marker proteins found in the analyzed genomes. (See attached Excel table)
